# Development of a clinical automatic calculation of hypoglycemia during hemodialysis risk in patients with diabetic nephropathy

**DOI:** 10.1186/s13098-023-01177-9

**Published:** 2023-10-13

**Authors:** Rui-Ting Zhang, Yu Liu, Ke-Ke Lin, Wan-Ning Jia, Quan-Ying Wu, Jing Wang, Xiao-Yan Bai

**Affiliations:** 1 School of Nursing, Beijing University of Chinese Medicine, Beijing, China; 2 Blood Purification Center of China-Japan Friendship Hospital, Beijing, China; 3 Nursing Department, Beijing Hospital, National Center of Gerontology, Institute of Geriatric Medicine, Chinese Academy of Medical Sciences, Beijing, China

**Keywords:** Diabetic nephropathy, Hemodialysis, Hypoglycemia, Risk prediction

## Abstract

**Background:**

Hypoglycemia is one of the most common complications in patients with DN during hemodialysis. The purpose of the study is to construct a clinical automatic calculation to predict risk of hypoglycemia during hemodialysis for patients with diabetic nephropathy.

**Methods:**

In this cross-sectional study, patients provided information for the questionnaire and received blood glucose tests during hemodialysis. The data were analyzed with logistic regression and then an automated calculator for risk prediction was constructed based on the results. From May to November 2022, 207 hemodialysis patients with diabetes nephropathy were recruited. Patients were recruited at blood purifying facilities at two hospitals in Beijing and Inner Mongolia province, China. Hypoglycemia is defined according to the standards of medical care in diabetes issued by ADA (2021). The blood glucose meter was used uniformly for blood glucose tests 15 minutes before the end of hemodialysis or when the patient did not feel well during hemodialysis.

**Results:**

The incidence of hypoglycemia during hemodialysis was 50.2% (104/207). The risk prediction model included 6 predictors, and was constructed as follows: Logit (*P*) = 1.505×hemodialysis duration 8~15 years (*OR* = 4.506, 3 points) + 1.616×hemodialysis duration 16~21 years (*OR* = 5.032, 3 points) + 1.504×having hypotension during last hemodialysis (*OR* = 4.501, 3 points) + 0.788×having hyperglycemia during the latest hemodialysis night (*OR* = 2.199, 2 points) + 0.91×disturbance of potassium metabolism (*OR* = 2.484, 2 points) + 2.636×serum albumin<35 g/L (*OR* = 13.963, 5 points)-4.314. The AUC of the prediction model was 0.866, with Matthews correlation coefficient (MCC) of 0.633, and Hosmer-Lemeshow *χ*^2^ of 4.447(*P* = 0.815). The automatic calculation has a total of 18 points and four risk levels.

**Conclusions:**

The incidence of hypoglycemia during hemodialysis is high in patients with DN. The risk prediction model in this study had a good prediction outcome. The hypoglycemia prediction automatic calculation that was developed using this model can be used to predict the risk of hypoglycemia in DN patients during hemodialysis and also help identify those with a high risk of hypoglycemia during hemodialysis.

**Supplementary Information:**

The online version contains supplementary material available at 10.1186/s13098-023-01177-9.

## Background

Diabetes nephropathy (DN) is the damage of renal function secondary to diabetes. It is one of the most important and serious complications of diabetes. When patients with DN progress to the end stage, they should be treated with renal replacement therapy, which usually includes hemodialysis [1]. Hemodialysis is of great significance to prolong the life span of the patients and improve their survival satisfaction [2]. According to the data in the Chinese National Renal Data System (CNRDS), 16.4% of patients with DN in China had received hemodialysis treatment by 2021, and DN has become the second cause of hemodialysis in China [3].

Hypoglycemia is one of the most common complications in patients with DN during hemodialysis, with an incidence rate of 53.5%~80% [4, 5]. When hypoglycemia during hemodialysis occurs in patients with DN, it not only affects the patients’ residual renal function and increases the risk of cognitive impairment, but also affects the quality of hemodialysis negatively and increases the risk of death [6, 7]. It is important for patients with DN to prevent hypoglycemia during hemodialysis. At present, research on hypoglycemia during hemodialysis in patients with DN mostly focuses on the effects of medical/nursing interventions [8–10], while little focuses on predicting the risk of hypoglycemia during hemodialysis. Early identification of the risk factors of hypoglycemia during hemodialysis in patients with DN and assessment of the risk of hypoglycemia in patients are of great significance for timely and effective prevention of hypoglycemia.

Point-of-care testing (POCT) is an effective method to detect and diagnose hypoglycemia during dialysis [11]. Blood glucose is generally measured 15 minutes before the end of hemodialysis in clinical practice [12]. However, since hemodialysis usually takes about 4 hours, routine blood glucose testing may not detect hypoglycemia effectively. Moreover, hypoglycemia symptoms during hemodialysis may overlap with the patients’ primary disease symptoms or hemodialysis reaction, which may make hypoglycemia more difficult to be detected on time. Even if hypoglycemia is detected in the patients during hemodialysis, the time-lag between detection and the treatment given is a concern. Therefore, it is necessary to construct a risk prediction model to anticipate the risk of hypoglycemia during hemodialysis in patients with DN. The model is expected to be used before hemodialysis by the medical staff to screen patients with high-level risk of hypoglycemia during hemodialysis and give early warning, which is helpful in implementing preventive intervention for them. However, such prediction models are lacking.

Thus, we compiled a questionnaire, based on a literature review, to investigate hypoglycemia during hemodialysis and identify its prediction factors in patients with DN. Subsequently, the logistic regression method was used to construct a risk prediction model for these patients of developing hypoglycemia during hemodialysis, then the model was the basis for the automatic calculation to predict the risk of hypoglycemia during hemodialysis in patients with DN.

The clinical predictive risk calculator for hypoglycemia in hemodialysis patients with DN constructed in this study aims to achieve a simple, low-cost, and personalized hypoglycemia risk prediction, transforming the treatment approach of intervention after the occurrence of hypoglycemia symptoms into targeted prevention of hypoglycemia, and improving the effectiveness of blood sugar management.

## Methods

This prediction model study is reported in accordance with the TRIPOD checklist [13]. The STROBE checklist was used to guide the submission [14].

### Participants

From May to November 2022, hemodialysis patients with DN were recruited by convenience sampling in blood purification centers of two hospitals in Beijing and Inner Mongolia province, China.

Inclusion criteria: ① being diagnosed with type 2 diabetes [15]; ② being diagnosed with DN according to nomenclature used by KDIGO [12];③ ≥18 years old; ④ received hemodialysis maintenance treatment for more than one year.

Exclusion criteria: ① received emergency hemodialysis currently;② combined with other serious diseases, such as severe heart failure, liver/renal insufficiency, respiratory failure and malignant tumor; ③ had history of kidney transplantation or liver transplantation; ④ language communication barriers; ⑤ being unable to complete the questionnaire even with assistance.

The study was approved by the Ethics Committee of Beijing University of Chinese Medicine.

### Developing a screening questionnaire on the predictors identified as probable causes of hypoglycemia during hemodialysis

#### Literature retrieval

Original literature on hypoglycemia in patients with DN undergoing hemodialysis was retrieved. Five subject terms–“diabetes”, “diabetic nephropathy”, “hemodialysis”, “hypoglycemia” and “influencing factor/related factor/ prediction factor/ predictor “-- were used to search English databases including PubMed, Cochrane Library, JBI、EMBASE, Wiley Online Library, Web of Science, and ProQuest Database, and Chinese databases including the Chinese Journal Full-text Database (CJFD), Wan fang Database, VIP Chinese Science and Technology Journal Full-text Database, and China Biomedical Abstract Database. The inclusion criteria were: (1) literature investigated factors related to hypoglycemia during hemodialysis; (2) literature involved patients with type 2 diabetes; (3) literature available for retrieval from the inceptions of the database to March 31, 2022; and (4) The language is Chinese or English. Duplicates were excluded. Two researchers read the titles and abstracts independently, and selected literature according to the inclusion and exclusion criteria, then merged their selected qualified literature. Disagreements during the literature selection were dissolved with a third senior researcher.

A total of 232 pieces of literature were retrieved, and the literature was screened according to the content on the predictors of hypoglycemia in patients with DN during hemodialysis. Finally, 28 articles were included in the study, including 12 RCTs, 5 cross-sectional studies, 2 case-control studies and 9 cohort studies.

#### Literature quality appraisal

Select corresponding quality evaluation tools based on the research type for literature quality appraisal. The quality of cross-sectional studies was assessed by using the American Agency for Health Care and Quality (AHRQ) methodological inventory of cross-sectional/prevalence studies. The quality of case-control studies and RCTs was assessed by using the National Institutes of Health Quality Assessment Tool, and the quality of cohort studies was assessed by using the Newcastle-Ottawa scale [16].

After literature quality assessment, 12 articles were determined to be of high quality.

#### Questionnaire development

We extract factors related to the risk of hypoglycemia with a frequency of ≥ 1 from the 12 articles and developed the “screening questionnaire on the factors related to hypoglycemia during hemodialysis.” Twenty factors related to hypoglycemia during hemodialysis in these 12 articles were extracted and included in the “Screening questionnaire on the factors related to hypoglycemia during hemodialysis”.

The questionnaire included four parts, with a total of 20 factors: ① demographic data: gender, age, educational background, and marital status; ② hemodialysis conditions: duration of hemodialysis, whether the hemodialysis was terminated in advance during the past month, vascular access condition, whether having eaten during hemodialysis, and whether having hypotension during last hemodialysis; ③ diabetes condition: duration of diabetes, current medication status of diabetes, whether received health education on hypoglycemia, whether measuring blood glucose regularly, and whether having hyperglycemia during the latest hemodialysis night (as a routine, patients are required to measure their blood glucose 19:00~24:00 on the night of hemodialysis); ④ laboratory examination indexes: blood potassium, blood phosphorus, hemoglobin, serum creatinine, serum albumin and parathyroid hormone levels in the past month. The first three parts were filled in by the patients, and the fourth part was filled in by the researchers.

### Sample size

According to the principle of events per variable [17], 5~10 patients for each independent variable are needed in Logistic analysis. Therefore, the sample size required for this study ranged from 100 to 200 cases (20 variables in this study). Taking a 10% loss of follow-up into account, the sample size needed for this study ranged from 111 to 223 patients. A total of 207 hemodialysis patients with DN participated in the study.

### Data collection

#### Questionnaire survey

The patients with DN were invited to participate in the study when they came to the blood purification center. After giving their informed consent, the patients were asked to fill in the “screening questionnaire on the factors related to hypoglycemia during hemodialysis” after finishing hemodialysis. For those who could not read the questionnaire, the researchers read the questionnaires for them. All questionnaires were collected by the researchers immediately upon completion to check for any missing items. If there are any missing items, the patient will be reminded to fill them out. Questionnaires with the number of missing items exceeding 10% of the total and those with obvious logic errors were excluded. After the patient filled in the first three parts of the questionnaire, the researcher filled in the fourth part by checking the patient’s electronic medical record.

#### Blood glucose assessment

According to standards of medical care in diabetes (2021) issued by the American Diabetes Association (ADA), hypoglycemia during hemodialysis in patients with DN can be divided into three levels: the first level of hypoglycemia is blood glucose < 70 mg/dl (3.9mmol/L), but ≥ 54 mg/dL (3.0 mmol/L); level 2 hypoglycemia, defined as blood glucose < 54 mg/dl (3.0mmol/l), is the threshold at which neuroglycopenic symptoms begin to occur and requires immediate action to resolve the hypoglycemic event; level 3 hypoglycemia is defined as a severe event characterized by altered mental and/or physical functioning that requires assistance from another person for recovery [14]. In this study, blood glucose < 70 mg/dl (3.9mmol/L) is regarded as suffering hypoglycemia during hemodialysis. The Sinocare safe + Code blood glucose meter was used for a blood glucose test 15 minutes before the end of hemodialysis or when the patient did not feel well (such as dizzy, sweating, weak) during hemodialysis.

### Statistical analysis

SPSS for Windows was used for the statistical analysis (version 21.0, IBM Corp, Armonk, United States). The continuous variables were presented as mean ± SD, whereas the categorical variables were presented as number and percentage. Continuous variables in the predictors were divided into categorical variables using the generalized additive model combined with professional knowledge, to facilitate clinical application. To test multicollinearity, the variance expansion factor was examined, followed by binary logistic regression analysis(LR) with forward selection to verify the contribution of the predictors to hypoglycemia during hemodialysis as well as to construct the prediction model. The Hosmer-Lemeshow goodness-of-fit test was used to determine the degree of calibration of the prediction model, and the area under the receiver operating characteristic curve (AUROC) was used to evaluate the discrimination of the prediction model, to calculate the sensitivity and specificity of the prediction model, and to verify the accuracy of the prediction model. Matthews correlation coefficient (MCC) was used to synthesize all indicators in the confusion matrix to reflect the comprehensive prediction effect of the model.

Then, the prediction model is the basis for the automatic calculation to predict the risk of hypoglycemia during hemodialysis in patients with DN. The scores of the predictors obtained by binary logistic regression were calculated according to their partial regression coefficient β [18]. β value of the predictors was expanded by 10 times each, and then divided by 5 to obtain assignment score rounding of each variable. The score of each predictor was added, and risk levels were determined based on the incidence of hypoglycemia [19]. A two-tailed P value < 0.05 was considered statistically significant.

## Results

### Samples

223 patients with DN and receiving hemodialysis were recruited. Sixteen patients refused to fill in the questionnaire due to privacy reasons. Finally, 207 patients finished the questionnaires, and all these questionnaires were valid, with the effective return rate of 92.8%.

207 hemodialysis patients with DN were 28~89 (63.09 ± 11.33) years old, the longest course of diabetes was 42 years, and the hemodialysis duration ranged from 1 to 21 (4.00 ± 3.74) years (Table 1). Of them, 104 experienced hypoglycemia during current hemodialysis, with the blood glucose ranging from 2.7 to 3.9 mmol/L. The incidence of hypoglycemia during hemodialysis in the study was 50.2%. 35 participants were tested for blood glucose 15 minutes before the end of hemodialysis, and 69 were tested because they did not feel well during hemodialysis.

**Table 1:** Characteristics of 207 hemodialysis participants with DN

Characteristics	*n*	%
Gender		
Male	142	68.6
Female	65	31.4
Educational background		
Primary school and below	149	71.9
Middle school	31	14.9
Secondary school	24	11.7
College and above	3	1.5
Having partner		
Yes	194	93.7
No	13	6.3
Duration of hemodialysis (year)		
1~4	39	18.8
5~7	19	9.2
8~15	74	35.8
16~21	75	36.2
Terminated hemodialysis in advance in the past month		
Yes	38	18.4
No	169	81.6
Vascular access condition		
Autologous fistula	174	84.1
Central cardiovascular	11	5.3
Central venous catheter	22	10.6
Having eaten during hemodialysis		
Yes	109	52.7
No	98	47.3
Having hypotension during last hemodialysis		
Yes	67	32.4
No	140	67.6
Current diabetes medication		
Oral hypoglycemic agents	30	14.5
Insulin	177	85.5
Whether received health education on hypoglycemia		
Yes	113	54.6
No	94	45.4
Whether measuring blood glucose regularly		
Yes	177	85.5
No	30	14.5
Having hyperglycemia during the latest hemodialysis night		
Yes	112	54.1
No	95	45.9
Blood potassium (mmol/L)		
Disturbance of potassium metabolism (<3.5 or >5.5)	114	55.1
3.5~5.5	93	44.9
Blood phosphorus (mmol/L)		
Disturbance of phosphorus metabolism (<0.81 or >1.45)	107	51.7
0.81~1.45	100	48.3
Hemoglobin (g/L)		
Anemia (Male<120; Female<110 )	112	54.1
Normal (Male ≥ 120; Female ≥ 110)	95	45.9
Serum creatinine (µmol/L)		
Male<53 or >106; Female<44 or >97	124	59.9
Male 53~106; Female 44~97	83	40.1
Serum albumin (g/L)		
<35	133	64.3
≥ 35	74	35.7
Intact parathyroid hormone (µg/L)		
<150 or >300	110	53.1
150~300	97	46.9

### Logistic regression prediction model construction

Binary logistic regression analysis was performed with the occurrence of hypoglycemia during hemodialysis as the dependent variable and 20 predictors mentioned above were taken as the independent variables. Variable assignments are shown in Table 2. There is no multicollinearity among independent variables (*VIF*<1.82). We found that hemodialysis duration 8 ~ 15 years, hemodialysis duration 16 ~ 21 years, having hypotension during last hemodialysis, having hyperglycemia on the latest hemodialysis night, disturbance of potassium metabolism and serum albumin < 35 g/L were predictors of hypoglycemia during hemodialysis for the patients with DN (Table 3).

**Table 2:** Variable assignment

Variable	Evaluation
Gender	Male = 0, Female = 1
Educational background	Primary school and below = 0, Middle school = 1, Secondary school = 2, College and above = 3
Having partner	No = 0, Yes = 1
Duration of hemodialysis	1~4 years = 000, 5~7 years = 100, 8~15 years = 010, 16~21 years = 001
Terminated hemodialysis in advance in the past month	No = 0, Yes = 1
Vascular access condition	Autologous fistula = 00, Central cardiovascular = 10, Central venous catheter = 01
Having eaten during hemodialysis	No = 0, Yes = 1
Having hypotension during last hemodialysis	No = 0, Yes = 1
Current diabetes medication	Hypoglycemic agents = 0, Insulin = 1
Whether received health education on hypoglycemia	No = 0, Yes = 1
Whether measuring blood glucose regularly	Irregularly = 0, Regularly = 1
Having hyperglycemia on the latest hemodialysis night	No = 0, Yes = 1
Blood potassium	3.5~5.5mmol/L = 0, Disturbance of potassium metabolism (<3.5mmol/L or >5.5mmol/L) = 1
Blood phosphorus	0.81~1.45mmol/L = 0, Disturbance of phosphorus metabolism (<0.81mmol/L or >1.45mmol/L) = 1
Hemoglobin	Male: ≥120 g/L = 0, Anemia (<120 g/L) = 1 Female: ≥110 g/L = 0, Anemia (<110 g/L) = 1
Serum creatinine	Male: 53~106µmol/L = 0, <53µmol/L or >106µmol/L = 1 Female: 44~97µmol/L = 0, <44µmol/L or >97µmol/L = 1
Serum albumin	≥ 35 g/L = 0, <35 g/L = 1
Intact parathyroid hormone	150~300µg/L = 0, <150 µg/L or >300 µg/L = 1
Hypoglycemia during hemodialysis	No = 0, Yes = 1

**Table 3:** Risk predictive model for hypoglycemia during hemodialysis

Variable	*β*	Standard error	Wald *χ*^2^ value	*P-*value	Odds ratio	95%*CI*
Constant	-4.314	0.728	35.129	<0.001	—	—
Hemodialysis duration 8~15 years	1.505	0.552	7.434	0.006	4.506	1.527~13.296
Hemodialysis duration 16~21 years	1.616	0.552	8.574	0.003	5.032	1.706~14.840
Having hypotension during last hemodialysis	1.504	0.452	11.075	0.001	4.501	1.856~10.917
Having hyperglycemia on the latest hemodialysis night	0.788	0.396	3.957	0.047	2.199	1.012~4.780
Disturbance of potassium metabolism	0.910	0.427	4.543	0.033	2.484	1.076~5.734
Serum Albumin<35 g/L	2.636	0.492	28.771	<0.001	13.963	5.328~36.589

Note: *β* is the regression coefficient

The regression equation of the model was obtained as follows: Logit(*P*) = 1.505×hemodialysis duration 8~15 years + 1.616×hemodialysis duration 16~21 years + 1.504×having hypotension during last hemodialysis + 0.788×having hyperglycemia on the latest hemodialysis night + 0.91×disturbance of potassium metabolism + 2.636×serum albumin<35 g/L -4.314.

### Model effect evaluation

**Fig. 1:**
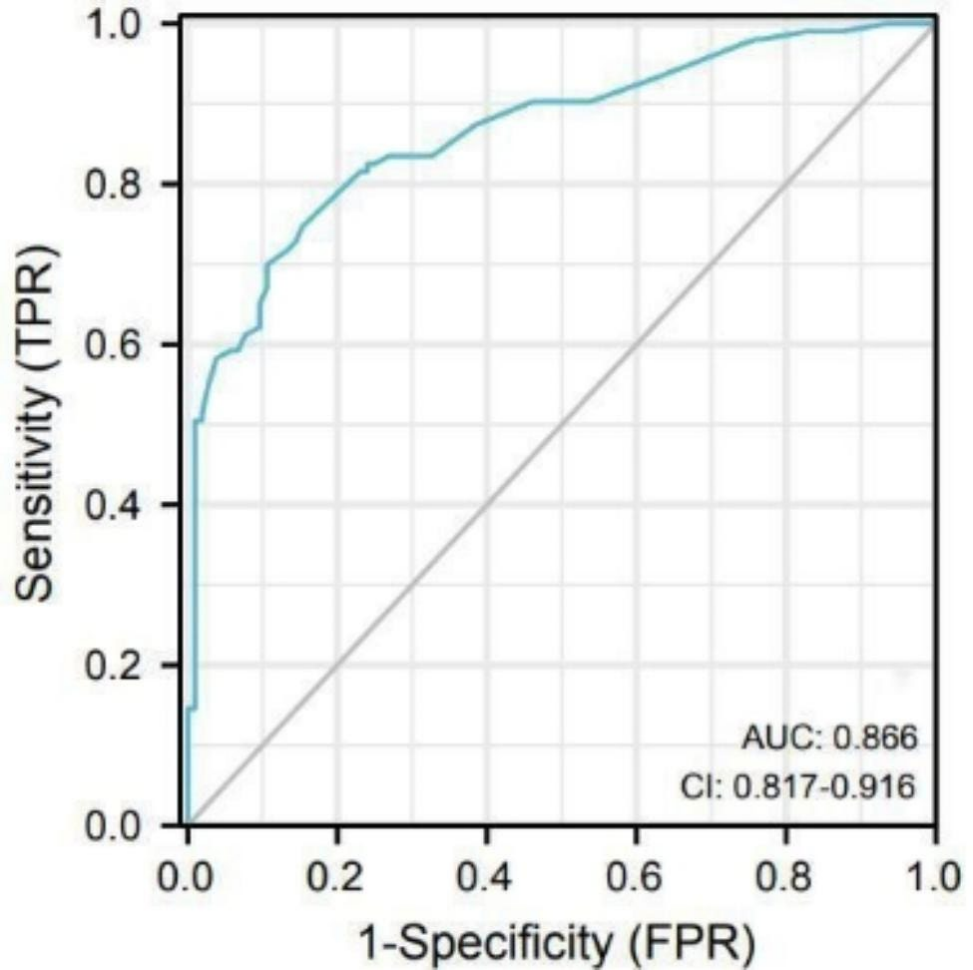
ROC curve for the Logistic regression model

After a goodness-of-fit test, the values of Hosmer-Lemeshow *χ*^2^ = 4.447 and *P* = 0.815 indicated that the model had excellent goodness of fit. The fitting effect between the occurrence of hypoglycemia predicted by the model and the actual occurrence of hypoglycemia during hemodialysis was examined by ROC curve (Fig. 1). The maximum value of Youden index was taken as the optimal critical value of the model. The area under the ROC curve was 0.866, the 95% CI was 0.817~0.916, the Youden index was 0.594, the optimal cutoff point was 0.495, and the sensitivity and specificity were 0.748 and 0.846, respectively.

### Risk automatic calculation construction

The risk automatic calculation of hypoglycemia during hemodialysis **(Annex 1)** was calculated according to the prediction regression equation. The six predictors obtained by binary logistic regression (Table 3) were rounded up according to their *β* value, and scored as 2, 3, 2, 5, 3 and 3 points, respectively. For instance, *β* value of serum albumin is 2.636, (2.636 × 10)/5 = 5.272. So serum albumin is rounded up to a core value of 5. The total score of the risk automatic calculation range is 0~18.

Based on the total scores, “Scoring sheet for predicting the risk of hypoglycemia during hemodialysis in patients with DN” was established(Table 4). The higher score indicates higher risk of hypoglycemia during hemodialysis in the patients with DN. The total scores of 14~18, 10~13, 6~9 and 0~5 respectively corresponded to the very high risk, high risk, moderate risk and low risk of hypoglycemia during dialysis [19]. The probability of hypoglycemia during hemodialysis was 84.9%, 66.3%, 26.8%, and 2.9%, respectively, for each group (very high risk, high risk, moderate risk and low risk).

**Table 4:** Risk assignment score during hemodialysis

Predictor		Fraction
Duration of hemodialysis	1~4 years	0
	5~7 years	0
	8~21 years	6
Having hypotension during last hemodialysis	Yes	3
	No	0
Having hyperglycemia on the latest hemodialysis night	Yes	2
	No	0
Blood potassium	<3.5mmol/L or >5.5mmol/L	2
	3.5~5.5mmol/L	0
Serum albumin	<35 g/L	5
	≥ 35 g/L	0

Risk of hypoglycemia during hemodialysis (total points):

Very high (14~18);High (10~13)≥ Moderate (6~9); Low (0~5)

## Discussion

In this study, Logistic regression was used to construct a risk prediction model of hypoglycemia during hemodialysis for the patients with DN, and the risk prediction automatic calculation was established based on the model. The calculation will help medical staff guide patients in medication and reduce the incidence of medical accidents. For patients, it will assist them to better understand their health state and take measures within their power to reduce the occurrence of hemodialysis hypoglycemia.

We found that the incidence of hypoglycemia during hemodialysis in the Chinese patients with DN was 50.2%, which was similar to the study of Gianchandani et al. [20] on maintenance hemodialysis patients with DN in the United States (51%). The patients with DN are more prone to hypoglycemia during hemodialysis compared with non-DN patients [21]. Among 207 patients, 69 patients with DN were diagnosed with hypoglycemia due to feeling discomfort in the unconventional blood glucose measurement time during hemodialysis, which suggests that the blood glucose monitoring time-point in the hemodialysis process of the patient needs further study, and it is necessary to predict the hypoglycemia risk of the patients before hemodialysis, to find the high-risk patients.

In this study, a risk prediction automatic calculation of hypoglycemia during hemodialysis in patients with DN was constructed. The risk prediction calculation allows medical staff predicting the risk of hypoglycemia in advance rather than taking intervention after hypoglycemia. We found there are six predictors of hypoglycemia during hemodialysis in the patients: hemodialysis duration 8 ~ 15 years, hemodialysis duration 16 ~ 21 years, having hypotension during last hemodialysis, having hyperglycemia on the latest hemodialysis night, disturbance of potassium metabolism, and serum albumin < 35 g/L. Among the predictors, serum albumin<35 g/L had the highest OR value. The risk of hypoglycemia during hemodialysis in the patients with hypoalbuminemia (serum albumin<35 g/L) was 13.963 times than that of the patients with normal-level serum albumin. It has been well recognized that serum albumin is an important index for evaluating the nutritional level of the body. In the case of long-term malnutrition, the concentration of serum albumin decreases [22]. Hypoalbuminemia in hemodialysis patients with DN indicates malnutrition, which may lead to reduced gluconeogenic matrix supply, inhibition of hepatic gluconeogenic capacity and insufficient glucose absorption, increasing the risk of hypoglycemia. During hemodialysis, glucose loss due to filtration further increases this risk [23, 24]. Previous reports have shown that serum albumin effectively predicted the mortality rate of maintenance hemodialysis patients after starting dialysis [25]. We also found the risk of hypoglycemia in hemodialysis patients with DN duration of 8~15 years and 16~21 years was 4.506 times and 5.032 times higher than that in patients 1~4 years, respectively. This indicates that the longer the course of DN, the higher the risk of hemodialysis hypoglycemia. Patients with 8~21 years of hemodialysis duration, having had a longer course of diabetes, often had adrenergic reaction defects [26], which led to the decrease of hypoglycemic symptom threshold, and subsequent delay of hypoglycemia detection during hemodialysis. Hypotension is one of the common complications in hemodialysis [27]. Gómez-Pulido et al. [28] have used machine learning classifiers to predict the risk of hypotension during hemodialysis. Our study indicated that the risk of hypoglycemia in the patients with hypotension during last hemodialysis was 4.501 times higher than in those without hypotension. The result was similar to the study of Maruyama et al. [29]. The occurrence of hypotension during last hemodialysis may cause nausea and vomiting, resulting in partial nutrient loss, which promotes hypoglycemia during hemodialysis [30]. This study showed that disturbance of potassium metabolism in the patients increases the risk of hyperglycemia during hemodialysis. When patients’ blood potassium is more than 5.5 mmol/L or less than 3.5mmol/L, the risk of hypoglycemia during hemodialysis is 2.484 times higher than in those with blood potassium being 3.5~5.5 mmol/L. In DN patients with hemodialysis, the increase in blood potassium is always accompanied by metabolic acidosis due to the compensatory regulation of cells, which inhibits gluconeogenesis [23], and increases the risk of hypoglycemia during hemodialysis. Hypokalemia, often manifested as gastrointestinal dysfunction, including nausea and vomiting, may increase the risk of hypoglycemia during hemodialysis. Additionally, glycogen synthesis requires the participation of K^+^. High blood potassium is conducive to glycogen synthesis [22], which reduces the concentration of glucose in blood and increases the risk of hypoglycemia during hemodialysis in the patients with DN. Siddiqa et al. [31] constructed a multivariable prognostic model for dialysis patients with end-stage renal disease and found a higher level of potassium, regardless of the albumin level, increased the mortality risk. Our study showed that patients with hyperglycemia on the latest hemodialysis night (19:00~24:00) had 2.199 times higher risk of hypoglycemia during hemodialysis than those without hyperglycemia. Such a blood glucose increase indicates unsatisfactory blood glucose control and great blood glucose fluctuations. Therefore, some patients received dose adjustments of hypoglycemic drugs (mainly incremental doses), which may enhance the risk of hyperglycemia during hemodialysis.

Previous studies have found that the use of hypoglycemic drugs like sulfonylureas and insulin may cause hypoglycemia during hemodialysis [32], but the hypoglycemic drugs did not enter the risk prediction model of hypoglycemia during hemodialysis for the patients with DN in this study. Since most sulfonylureas undergo renal metabolism, DN patients who enter the renal failure stage switch to insulin for hypoglycemic treatment under the doctor’s guidance. In this study, the majority (85.5%) of the patients with DN used insulin, only 14.5% of the patients used oral hypoglycemic drugs, which may be related to the fact that sulfonylureas did not enter the risk predictive model. Insulin was also not included in the risk predictive model, which may be related to more than half of the patients (54.1%) having hyperglycemia on the latest hemodialysis night, thereby intentionally reducing insulin dosage.

In this study, a risk automatic calculation of predicting hypoglycemia during hemodialysis in patients with DN was calculated according to the risk prediction model. The area under the ROC curve of the risk prediction model was 0.866, which shows that it has acceptable prediction effect [33]. The value of MCC was 0.633, indicating that the prediction result is close to the real result [34]. Medical staff can predict the risk of hypoglycemia during hemodialysis for the patients with DN by using the calculation to collect the patient’s hemodialysis duration, whether having hypotension during last hemodialysis, whether having hyperglycemia on the latest hemodialysis night, the blood potassium level, and the serum albumin level in the past month. The probability of hypoglycemia during hemodialysis in very high (score = 14~18), high (score = 10~13), moderate (score = 6~9), and low (score 0~5) categories was 84.85%, 66.29%, 26.83%, and 2.86%. Medical staff should pay attention to the patients with very high risk or high risk and conduct intervention for them, such as using sugary dialysate, arranging eating during hemodialysis, etc.

This study used a convenience sampling method, which may lead to the limitation in the conclusion. Secondly, due to the resources limit, we did not dynamically and continuously monitor the blood glucose during hemodialysis for the patients. Measuring fingertip blood glucose has certain limitations, but it is common in the current clinical practice. Therefore, the risk prediction automatic calculation has good operability and feasibility. Finally, the prediction model can be applied to patients whose hemodialysis duration is no greater than 21 years. Future studies should consider longer hemodialysis duration. In addition, because the candidate factors of the risk prediction model in this study were extracted from the literature, new candidate factors may be included with the deepening of research in this field. It is suggested that the literature search results should be regularly updated in the future to optimize the model.

This risk prediction calculation will help medical staff to predict the risk of hypoglycemia in hemodialysis patients before hemodialysis. Since the predictors in the calculation are routinely monitored in clinical practice and easy to obtain, it is convenient and fast for medical staff to use.

## Conclusions

The incidence of hypoglycemia during hemodialysis of patients with DN was 50.2%. The hemodialysis duration of 8 ~ 21 years, having hypotension during last hemodialysis, having hyperglycemia on the latest hemodialysis night, disturbance of potassium metabolism, and serum albumin < 35 g/L were the predictors for hypoglycemia during hemodialysis of the patients. The automatic calculation for hemodialysis patients with DN was constructed based on the logistic regression equation and has good predictive performance. In the automatic calculation, the total scores of 14~18, 10~13, 6~9, and 0~5, respectively corresponded to the very high, high, moderate, and low risk of hypoglycemia during hemodialysis. For the future research, it would be of great importance to explore other factors that may contribute to hypoglycemia prediction, analyze the long-term impact of our research findings, or investigate the effectiveness of different interventions to avoid hypoglycemia.

### Electronic supplementary material

Below is the link to the electronic supplementary material.

Supplementary Material 1

Supplementary Material 2

## Data Availability

The datasets used and/or analyzed during the current study are available from the corresponding author on reasonable request.

## Abbreviations

DN: Diabetes Nephropathy
CNRDS: Chinese National Renal Data System
POCT: Point-of-care Testing
CJFD: Chinese Journal Full-text Database
AHRQ: Agency for Healthcare Research and Quality
ADA: American Diabetes Association
LR: Logistic Regression Analysis
AUROC: The Area Under the Receiver Operating Characteristic Curve
