# Supplementary material for: Development of a clinical automatic calculation of hypoglycemia during hemodialysis risk in patients with diabetic nephropathy
Source: Diabetol Metab Syndr. 2023 Oct 13;15:199. doi: 10.1186/s13098-023-01177-9 (PMC10571353; doi:10.1186/s13098-023-01177-9)
Supplement: Supplementary file 2 — Supplementary Material 2 [file 13098_2023_1177_MOESM2_ESM.pdf]

Consent confirmatory letter

Written informed consent for patient information and images to be published was provided by the patients. Authors have obtained written informed consent and the written consent itself held by the authors.

Sunday, October 25th, 2022

内蒙古科技大学包头医学院第一附属医院
护理部
